# The role of metabolic score for visceral fat in the prediction of atrial fibrillation recurrence risk after catheter ablation

**DOI:** 10.3389/fendo.2025.1607497

**Published:** 2025-09-02

**Authors:** Yazhe Ma, Xiaolong Gao, Jianying Sun, Xiaohui Kuang, Xi Zhang, Feiyu Wei, Tao Ma, Yanju Cui, Jia Guo, Peng Wu, Jiangwen Liu, Jie Fan

**Affiliations:** ^1^ Yunnan Arrhythmia Research Center, Division of Cardiology, the First People’s Hospital of Yunnan Province, the Affiliated Hospital of Kunming University of Science and Technology, Kunming, China; ^2^ Department of Biopharmaceuticals and Tianjin Key Laboratory on Technologies Enabling Development of Clinical Therapeutics and Diagnostics, School of Pharmacy, Tianjin Medical University, Tianjin, China

**Keywords:** metabolic score of visceral fat, atrial fibrillation, visceral obesity, catheter ablation, independent risk factor

## Abstract

**Background:**

Previous studies show that visceral fat tissue (VAT) play an important role in atrial fibrillation (AF). The metabolic score of visceral fat (METS-VF), a new surrogate to estimate VAT, is associated with cardiovascular mortality risk. In this study, we try to investigate the association between METS-VF and the risk of AF recurrence after catheter ablation.

**Methods:**

478 consecutive patients underwent catheter ablation were obtained and used to assess the relationship between METS-VF and the risk of AF recurrence. Cox regression was used to calculate the hazard ration (HR) of METS-VF for the risk of AF recurrence. Restricted cubic splines (RCS) was used to assessed the linear relationship between METS-VF and the AF recurrence risk.

**Results:**

A total of 112(23.4%) patients experienced AF recurrence during 18.0 ± 9.6 months follow-up. The AF recurrence rate was significantly higher in the highest quartile of METS-VF than the other three quartiles (log rank = 0.021). In the univariate cox regression, LAD, and MET-VF were associated with AF recurrence (p<0.0001). In the multiple Cox regression results, compared with the participants with lowest METS-VF (Q1), the hazard ratio (HR) (95% CI) for the AF recurrence risk was 1.29 (0.73, 2.29) for Q2 (p=0.39), 1.59 (0.88 – 2.87) for Q3 (p=0.12), and 2.22 (1.20, 4.12) for Q4 (p<0.01) respectively.

**Conclusions:**

METS-VF was positively associated with the elevated AF recurrence risk. Our findings show that the METS-VF could be used to AF recurrence risk stratification.

## Graphical Abstract

This is the first study investigating the relationship between visceral adipose tissue (VAT) surrogates, such as lipid accumulation product (LAP), cardiometabolic index (CMI), and metabolic score for visceral fat (METS-VF), and atrial fibrillation (AF) recurrence following ablation. METS-VF, a reliable surrogate reflecting the VAT, was positively associated with elevated AF recurrence after ablation. METS-VF could be used to screen individuals with an elevated risk of AF recurrence in clinical practice.

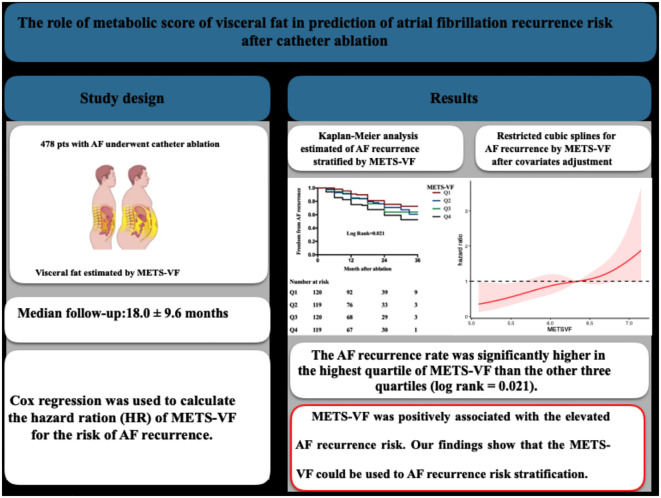

## Introduction

Atrial fibrillation (AF) is the most common sustained arrhythmia, affecting over 33 million people worldwide (1, 2). It significantly increases the risk of stroke, heart failure, cardiovascular hospitalization, and all-cause mortality (3). Although catheter ablation is an effective rhythm control strategy, AF recurrence remains common, with rates ranging from 24% to 39% (4, 5). Identifying robust predictors of recurrence is crucial for improving long-term outcomes after ablation. Among the known risk factors, obesity—in particular the accumulation of visceral adipose tissue (VAT)—has been increasingly recognized as a key contributor to the development and progression of AF (6). However, direct imaging of VAT via CT, MRI, or dual-energy X-ray absorptiometry (DXA) is often impractical in routine clinical settings due to cost and limited accessibility (7, 8). To overcome this limitation, several surrogate indices have been proposed, including the lipid accumulation product (LAP), the cardiometabolic index (CMI), and the metabolic score for visceral fat (METS-VF). Unlike traditional markers, METS-VF integrates multiple dimensions of metabolic health—such as insulin resistance, BMI, and lipid metabolism—to provide a more comprehensive estimation of visceral fat burden (9). However, the association between METS-VF and AF recurrence after ablation remains largely unexplored. In this study, we aimed to investigate the predictive value of METS-VF for AF recurrence following catheter ablation. In addition, we compared its performance with those of other VAT-related indicators [i.e., LAP, CMI, and the metabolic score for insulin resistance (METS-IR)] using time-dependent receiver operating characteristic (ROC) curves to determine whether METS-VF offers superior prognostic utility.

## Methods

### Study population

We enrolled 478 patients who underwent the first radiofrequency catheter ablation for AF between January 2021 and March 2024 at the First People’s Hospital of Yunnan Province (Kunming, China). The exclusion criteria were as follows: 1) moderate-to-severe valvular disease; 2) uncontrolled thyroid dysfunction; 3) left atrial thrombosis; 4) acute coronary syndrome, myocardial infarction, and cardiac surgery in the previous 3 months; 5) contraindication of anticoagulation; 6) pregnancy; 7) hepatic or renal failure; and 8) patients who died or who were lost to follow-up. The study is in compliance with the principles of the Declaration of Helsinki and was approved by the Ethics Committee of Yunnan First People’s Hospital.

### Data collection and definitions

Clinical data including age, sex, BMI, waist circumference, hypertension (HT), diabetes mellitus (DM), history of stroke, heart failure (HF), and type of AF (paroxysmal AF or persistent AF) were collected. The CHA_2_DS_2_-VASc score was calculated for each patient. Serum blood biomarkers such as fasting plasma glucose (fGLU), creatinine, total cholesterol (TC), low-density lipoprotein cholesterol (LDL-C), and high-density lipoprotein cholesterol (HDL-C) were determined. Transthoracic echocardiography was employed to record the left atrial diameter (LAD) and the left ventricular ejection fraction (EF). The VAT surrogates were calculated as follows: BMI = weight (kg)/height^2^ (m^2^); WHtR = waist (cm)/height (cm); LAP(women) = TG (mmol/L) * [WC (cm) − 58]; LAP(men) = TG (mmol/L) * [WC (cm) − 65]; CMI = TG (mmol/L)/HDL-C (mmol/L) * WHtR; METS-IR = Ln[2 × FPG (mg/dl) + TG (mg/dl)] × BMI (kg/m^2^)/Ln [HDL-C (mg/dl); and METS-VF = 4.466 + 0.011 × [Ln(METS-VF)]^3^ + 3.239 × [Ln(WHtR)]^3^ + 0.319 × Sex (men = 1, women= 0) + 0.594 × [Ln(Age) (years)] (10).

### Electrophysiology study and catheter ablation

The details regarding electrophysiology and catheter ablation are available in our previous study (11). In brief, an open irrigated catheter (ST Catheter, Biosense-Webster, Diamond Bar, CA, USA) was used to perform circumferential pulmonary vein (PV) isolation. Additional ablations in the superior vena cava or other non-PV triggers were performed when mappable AF triggers were available.

### Follow-up

Patients were followed up at the outpatient department at 3, 6, 9, and 12 months after ablation and every 6 months thereafter, for a medium follow-up duration of 18 ± 9.6 months. At each visit, a 12-lead electrocardiogram and 24-h Holter monitoring were performed. When a patient showed symptoms of palpitations, the Holter data were obtained to evaluate arrhythmia. AF recurrence was defined as any episode of AF or atrial tachycardia lasting more than 30 s.

### Statistical analysis

Descriptive statistics are reported as frequencies and percentages for categorical variables and as medians with interquartile ranges (Q1–Q3) for continuous variables. Continuous variables were compared using the Wilcoxon rank-sum test or the Kruskal–Wallis test, while categorical variables were compared using the chi-square test. Kaplan–Meier survival curves were generated to visualize the time to AF recurrence stratified by the METS-VF quartiles, and differences between groups were assessed using the log-rank test. The association between the clinical/metabolic variables and AF recurrence was assessed using Cox proportional hazards regression. Univariable Cox models were performed first, followed by multivariable models adjusting for potential confounders. The final multivariate model included age, sex, BMI, stroke, HT, HF, cardiovascular disease (CVD), DM, TC, LDL-C, HDL-C, triglycerides (Tg), fGLU, and LAD. Restricted cubic spline (RCS) modeling was used to explore the dose–response relationship and the potential non-linearity between METS-VF and AF recurrence risk. Time-dependent ROC curve analysis was conducted at 12, 24, and 36 months to evaluate and compare the predictive performance of METS-VF, CMI, LAP, and METS-IR. The area under the curve (AUC) values were compared using DeLong’s test. All data analyses were performed using R or SPSS. A *p* < 0.05 was considered statistically significant.

## Results

### Baseline characteristics

This study included 478 patients (age, 58.9 ± 10.6 years; 62.8% men) with either paroxysmal (*n* = 370) or persistent AF (*n* = 108). The demographic characteristics, the clinical and laboratory data, and the VAT surrogates are summarized in **Table 1**. After a mean follow-up period of 18.0 ± 9.6 months, AF recurrence was observed in 112 (23.4%) patients. As shown in **Table 1**, patients with AF recurrence exhibited larger LAD (39.4 ± 5.7 *vs*. 37.4 ± 5.9, *p* = 0.002), higher CMI (0.68 *vs*. 0.56, *p* = 0.039), higher LAP (28.1 *vs*. 23.5, *p* = 0.023), higher MET-IR (34.29 ± 7.68 *vs*. 32.52 ± 6.94, *p* = 0.02), and higher METS-VF (6.38 ± 0.50 *vs*. 5.99 ± 0.53, *p* = 0.004) compared to patients without AF recurrence. Furthermore, patients were stratified into four groups according to the METS-VF quartiles (**Table 2**). The quartile thresholds for METS-VF were determined as 5.90, 6.35, and 6.68. Patients in the highest quartile of METS-VF had higher BMI, larger LAD, and higher fGLU, CMI, LAP, and METS-IR than those in the other three groups (all *p* < 0.0001).

**Table 1:** Baseline characteristics of patients.

Parameter	Total (*n* = 478)	Recurrence (+) (*n* = 112)	Recurrence (−) (*n* = 366)	*p*
Age (years)	58.9 ± 10.6	57.8 ± 11.2	58.9 ± 10.6	0.366
Men	300 (62.8%)	76 (67.3%)	224 (61.4%)	0.258
BMI (kg/m^3^)	24.89 ± 3.47	25.26 ± 3.52	24.77 ± 3.46	0.373
History of CAD	51 (10.7%)	12 (10.4%)	39 (10.7%)	0.925
Diabetes mellitus	47 (9.8%)	14 (12.2%)	33 (9.1%)	0.333
Hypertension	221 (46.2%)	59 (51.3%)	162 (44.6%)	0.211
Stroke	45 (9.4%)	7 (6.1%)	38 (10.5%)	0.161
Persistent AF	108 (22.6%)	31 (27.0%)	77 (21.2%)	0.199
LAD (mm)	37.9 ± 5.9	39.4 ± 5.7	37.4 ± 5.9	0.002*
EF	65.2 ± 15.4	63.7 ± 6.6	65.6 ± 17.6	0.270
CHA_2_DES_2_-VASc	1 (1–3)	1 (1–3)	1 (1–3)	0.655
fGLU	5.0 (4.5–5.5)	5.0 (4.5–5.4)	5.0 (4.5–5.7)	0.473
eGFR	92.4 (80.6–101.0)	92.8 (77.5–103.3)	92.3 (81.1–100.3)	0.627
CMI	0.58 (0.39–0.93)	0.68 (0.44–1.09)	0.56 (0.38–0.87)	0.039*
LAP	24.3 (14.4–39.7)	28.1 (17.4–42.1)	23.5 (13.8–36.1)	0.023*
METS-IR	32.94 ± 7.16	34.29 ± 7.68	32.52 ± 6.94	0.02*
METS-VF	6.27 ± 0.54	6.38 ± 0.50	5.99 ± 0.53	0.004*

Data are median (interquartile range), mean ± SD, or *n* (%).

*BMI*, body mass index; *CAD*, coronary artery disease; *AF*, atrial fibrillation; *LAD*, left atrial diameter; *EF*, ejection fraction; *fGLU*, fasting plasma glucose; *eGFR*, estimated glomerular filtration rate; *CMI*, cardiometabolic index; *LAP*, lipid accumulation product; *METS-IR*, metabolic score for insulin resistance; *METS-VF*, metabolic score for visceral fat. * P<0.05.

**Table 2:** Clinical characteristics according to the METS-VF quartiles.

Variable	Q1 (*n* = 120	Q2 (*n* = 119)	METS-VF	Q4 (*n* = 119)	*p*
Age (years)	55.3 ± 11.9	58.9 ± 10.3	60.5 ± 10.1	58.6 ± 10.7	0.001
Men	68 (56.7%)	74 (62.2%)	75 (62.5%)	83 (69.7)	0.22
BMI (kg/m^3^)	23.31 ± 2.97	23.97 ± 3.09	24.97 ± 3.03	27.31 ± 3.41	<0.0001
History of CAD	11 (9.1%)	15 (12.6%)	13 (10.8%)	12 (10.1%)	0.85
Diabetes mellitus	8 (6.7%)	9 (7.6%)	18 (15.1%)	12 (10.1%)	0.129
Hypertension	47 (39.2%)	55 (46.2%)	57 (47.5%)	55 (46.2%)	0.247
Stroke	7 (5.8%)	15 (12.6%)	12 (10.0%)	11 (9.2%)	0.351
Persistent AF	33 (27.5%)	31 (26.1%)	21 (17.5%)	21 (17.6)	0.174
LAD (mm)	36.1 ± 5.9	37.1 ± 5.4	38.3 ± 5.1	40.3 ± 6.2	<0.0001
EF	67.3 ± 16.4	66.9 ± 17.5	62.8 ± 7.2	63.6 ± 6.8	0.064
CHA_2_DES_2_ **-**Vasc	1 (1–2)	2 (0–3)	2 (1–3)	1 (1–3)	0.060
fGLU	4.8 (4.4–5.2)	4.8 (4.4–5.3)	5.3 (4.6–5.8)	5.1 (4.7–5.6)	<0.0001
EGFR	95.8 (81.4–104.2)	91.6 (80.6–99.8)	91.85 (79.1–100.0)	92.1 (82.0–98.9)	0.278
CMI	0.47 (0.31–0.71)	0.52 (0.34–0.93)	0.64 (0.43–0.94)	0.76 (0.49–1.1)	<0.0001
LAP	12.0 (7.3–19.5)	20.5 (12.6–29.2)	27.8 (21.2–43.8)	39.7 (27.1–56.2)	<0.0001
METS-IR	30.03 ± 6.19	31.35 ± 6.25	33.40 ± 6.75	37.01 ± 7.46	<0.0001
AF recurrence	18.3%	22.7%	22.5%	32.8%	0.061

Data are median (interquartile range), mean ± SD, or *n* (%).

*BMI*, body mass index; *CAD*, coronary artery disease; *AF*, atrial fibrillation; *LAD*, left atrial diameter; *EF*, ejection fraction; *fGLU*, fasting plasma glucose; *eGFR*, estimated glomerular filtration rate; *CMI*, cardiometabolic index; *LAP*, lipid accumulation product; *METS-IR*, metabolic score for insulin resistance; *METS-VF*, metabolic score for visceral fat.

### Cox regression and Kaplan–Meier analyses

**Figure 1:**
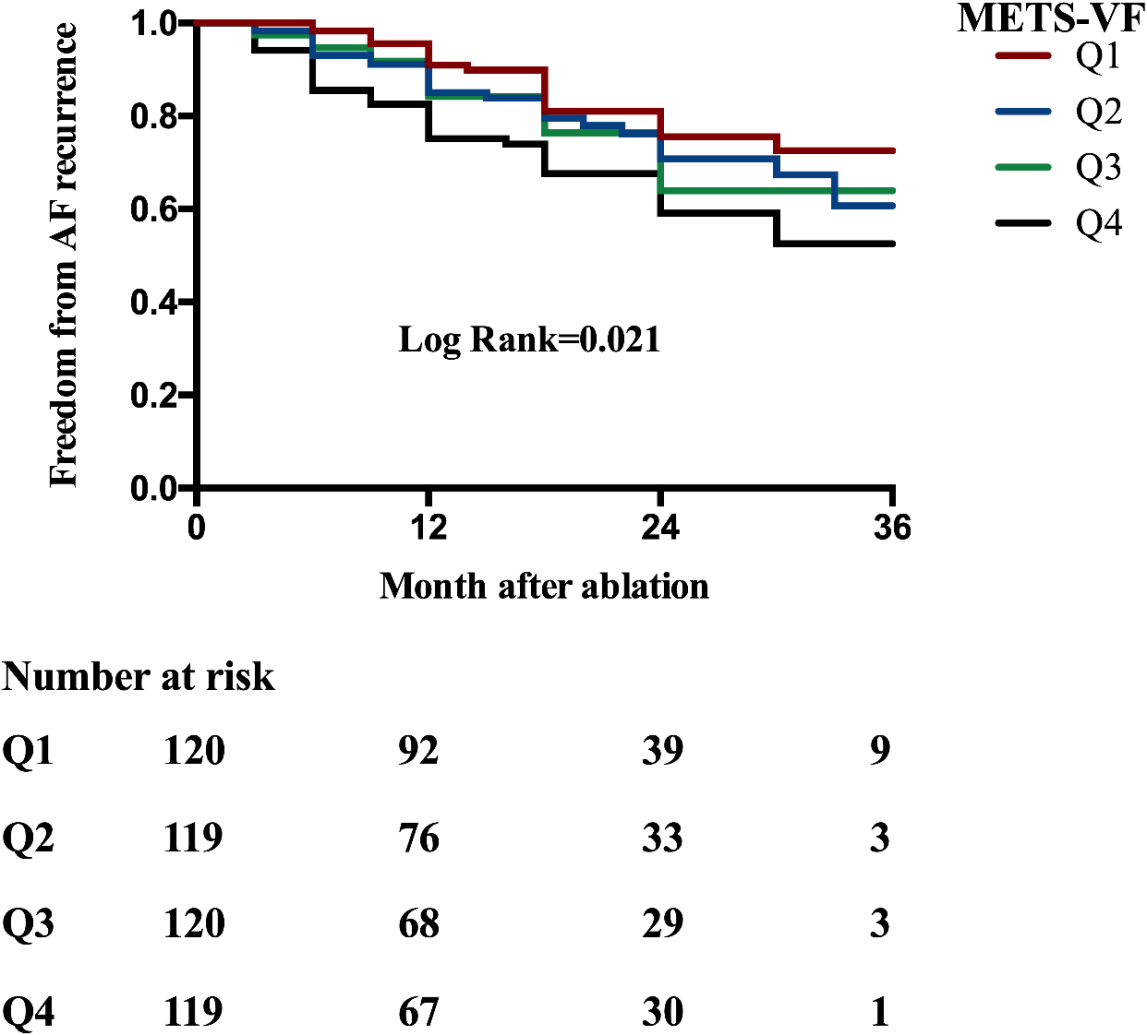
Kaplan–Meier analysis estimating AF recurrence in patients with AF undergoing catheter ablation stratified by the METS-VF quartiles (overall log-rank = 0.021). *AF*, atrial fibrillation; *METS-VF*, metabolic score for visceral fat.

Kaplan–Meier analysis was performed to estimate the time to AF recurrence after catheter ablation across the different METS_VF risk strata (**Figure 1**). Kaplan–Meier analysis revealed a significant difference in AF recurrence across the METS-VF quartiles (log-rank *p* = 0.021), with higher METS-VF scores associated with an increased risk of recurrence following catheter ablation. Cox proportional hazards regression models were then utilized to identify whether METS-VF and other metabolic indices were independently associated with AF recurrence over time. In the univariable Cox regression analysis (**Table 3**), several clinical and metabolic variables were assessed for their association with AF recurrence following catheter ablation. Among them, LAD and a higher METS-VF were independently associated with the recurrence of AF after ablation. Multivariable Cox regression (**Table 3**) confirmed METS-VF as an independent predictor of AF recurrence after adjusting for confounders. To further evaluate risk stratification, METS-VF was analyzed as a categorical variable using quartiles (**Table 4**). In all three adjustment models, patients in the highest quartile (Q4) showed a significantly elevated risk of AF recurrence compared with those in Q1.

**Table 3:** Univariable Cox regression analysis to predict AF recurrence after ablation.

Variable	Univariate model			Multivariate model		
	HR	95%Cl	*p*	HR	95%Cl	*p*
Age >65 years	1.22	0.83–1.79	0.312	1.05	0.70–1.56	0.816
Men	1.21	0.82–1.79	0.332	1.07	0.72–1.60	0.734
BMI (kg/m^3^)	1.03	0.98–1.09	0.249	0.97	0.91–1.03	0.358
History of CAD	1.21	0.66–2.21	0.533			
Diabetes mellitus	1.22	0.70–2.14	0.482			
Hypertension	1.26	0.87–1.81	0.218			
Stroke	0.68	0.32–1.47	0.328			
Persistent AF	1.21	0.80–1.83	0.358	1.22	0.80–1.84	0.356
LAD (mm)	1.05	1.02–1.08	0.003*	1.04	1.00–1.07	0.029*
EF	0.11	0.01–1.41	0.089			
CHA_2_DES_2_-Vasc	1.02	0.90–1.16	0.749			
fGLU	1.10	0.95–1.28	0.190			
EGFR	1.00	0.99–1.01	0.745			
Cardiometabolic index	1.14	0.93–1.42	0.209			
LAP	1.00	0.99–1.01	0.212			
METS-IR	1.03	0.99–1.05	0.058			
METS-VF	1.78	1.25–2.54	0.001*	1.73	1.14–2.61	0.01*

*BMI*, body mass index; *CAD*, coronary artery disease; *AF*, atrial fibrillation; *LAD*, left atrial diameter; *EF*, ejection fraction; *fGLU*, fasting plasma glucose; *eGFR*, estimated glomerular filtration rate; *CMI*, cardiometabolic index; *LAP*, lipid accumulation product; *METS-IR*, metabolic score for insulin resistance; *METS-VF*, metabolic score for visceral fat.

**Table 4:** Multivariable cox regression analysis to predict AF recurrence after ablation.

	Model 1		Model 2		Model 3	
	HR (95%CI)	*p*-value	HR (95%CI)	*p*-value	HR (95%CI)	*p*-value
METS-VF	1.92 (1.26–2.94)	<0.01	2.07 (1.34–3.19)	<0.01	2.02 (1.29–3.14)	<0.01
METS-VF						
Q1 (4.53–5.9]	1.00 (Ref)		1.00 (Ref)		1.00 (Ref)	
Q2 (5.9–6.35]	1.27 (0.72–2.24)	0.41	1.30 (0.73–2.30)	0.37	1.24 (0.69–2.20)	0.47
Q3 (6.35–6.678]	1.51 (0.84–2.69)	0.17	1.56 (0.87–2.81)	0.14	1.52 (0.84–2.76)	0.17
Q4 (6.678–7.35]	2.14 (1.18–3.88)	0.01	2.27 (1.25–4.13)	0.01	2.16 (1.17–3.99)	0.01

Model 1 was adjusted for age, gender, and BMI. Model 2 is model 1 adjusted for stroke, hypertension, heart failure, cardiovascular disease (CVD), and diabetes mellitus (DM). Model 3 is model 2 adjusted for total cholesterol, low-density lipoprotein (LDL), high-density lipoprotein (HDL), triglycerides (Tg), and fasting plasma glucose (fGLU).

*HR*, hazard ratio; *95%CI*, 95% confidence interval; *METS-VF*, metabolic score for visceral fat.

### Predictive performance of METS-VF *vs*. comparator models

**Figure 2:**
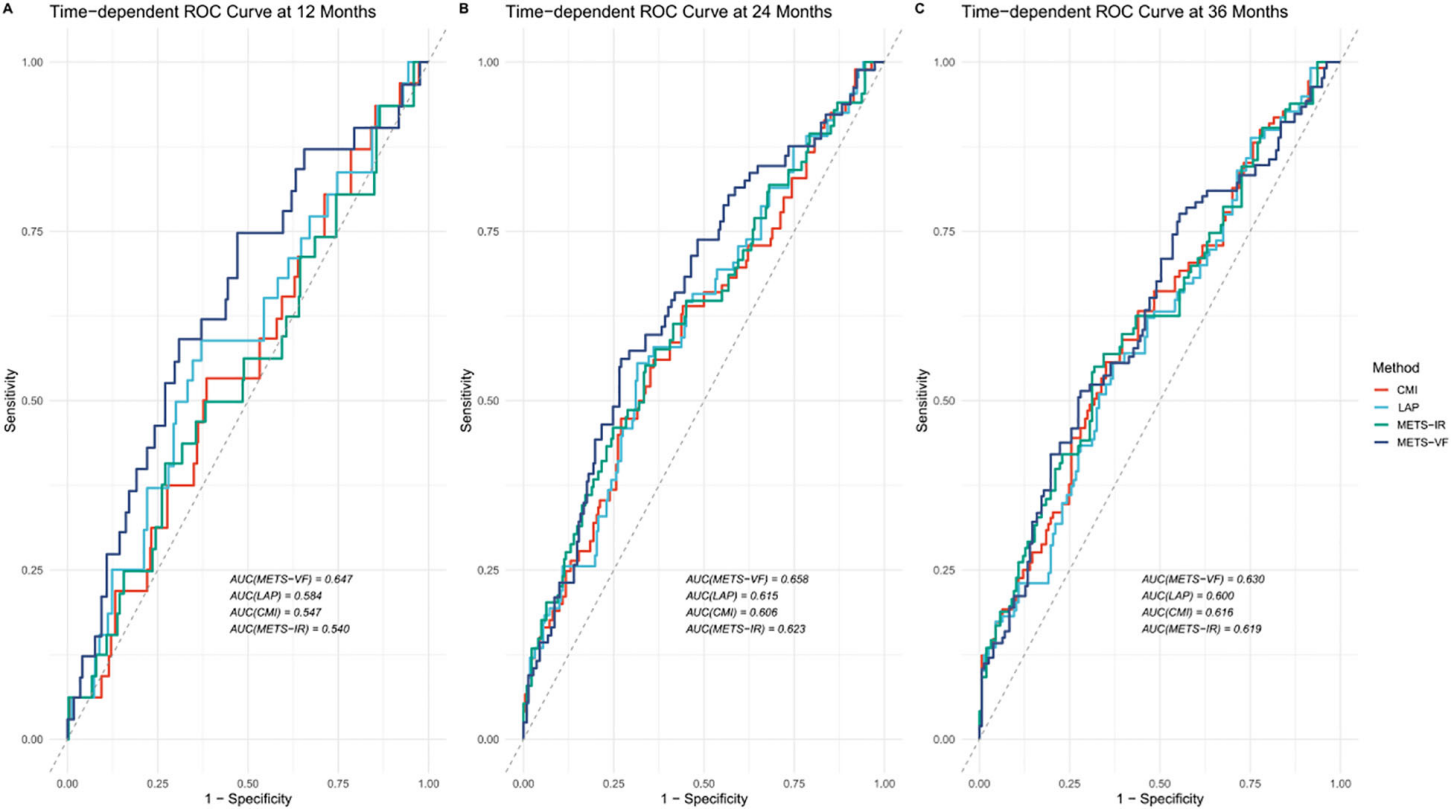
Time-dependent receiver operating characteristic (ROC) curves for predicting atrial fibrillation (AF) recurrence after catheter ablation. *METS-VF*, metabolic score for visceral fat; *LAP*, lipid accumulation product; *CMI*, cardiometabolic index; *METS-IR*, metabolic score for insulin resistance. **(A)** ROC curves for predicting AF one- year recurrence after catheter ablation; **(B)** ROC curves for predicting AF two- year recurrence after catheter ablation; **(C)** ROC curves for predicting AF three year recurrence after catheter ablation.

To evaluate the discriminative ability of METS-VF in predicting AF recurrence after catheter ablation, time-dependent ROC curve analyses were conducted and the results compared against three established metabolic indices: CMI, LAP, and METS-IR. **Figure 2** shows the time-dependent ROC curves generated at 12, 24, and 36 months of follow-up. The corresponding AUC values are presented in **Table 5**. Comparisons were performed using DeLong’s test to assess statistical significance. At the 12-month mark, METS-VF demonstrated significantly better predictive performance than LAP (*p* = 0.027), CMI (*p* = 0.003), and METS-IR (*p* = 0.013). However, the differences in the AUCs between METS-VF and the comparator models at 24 and 36 months were not statistically significant (all *p* > 0.3).

**Table 5:** Comparison of the predictive performance of METS-VF and the comparator models at different time points.

Model comparison	Month	AUC (METS-VF)	AUC (comparator)	*p*-value (DeLong)
METS-VF *vs*. LAP	12	0.677	0.618	0.027*
	24	0.659	0.635	0.328
	36	0.655	0.629	0.275
METS-VF *vs*. CMI	12	0.677	0.572	0.003**
	24	0.659	0.626	0.305
	36	0.655	0.634	0.504
METS-VF *vs*. METS-IR	12	0.677	0.582	0.013*
	24	0.659	0.625	0.302
	36	0.655	0.634	0.511

AUC, area under the curve; METS-VF, metabolic score for visceral fat; METS-IR, metabolic score for insulin resistance, *LAP*, lipid accumulation product; *CMI*, cardiometabolic index.* P<0.05.

### Dose–response relationship between METS-VF and AF recurrence

**Figure 3:**
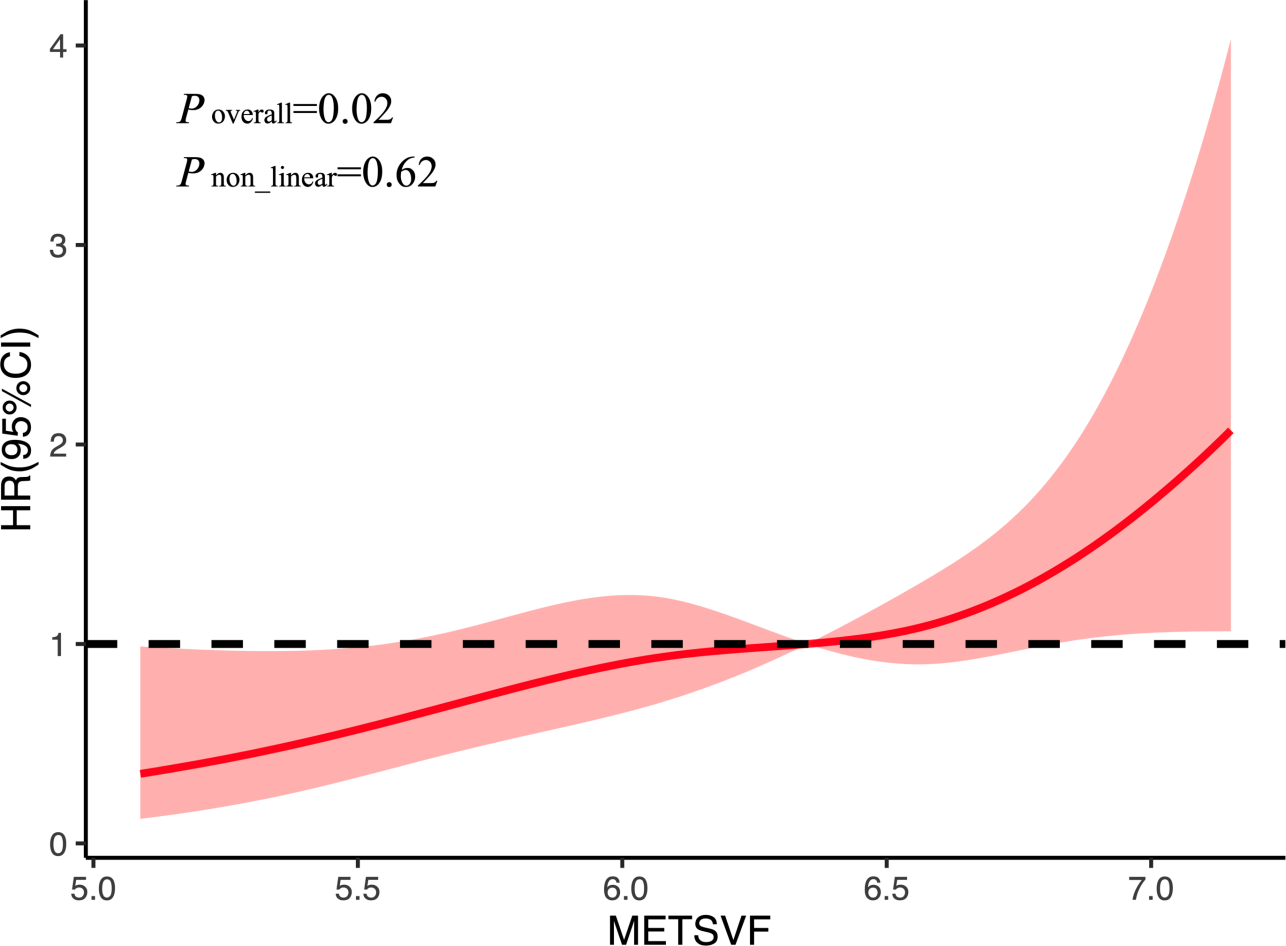
Restricted spline curves for AF recurrence by METS-VF after adjustment for covariates. *AF*, atrial fibrillation; *METS-VF*, metabolic score for visceral fat; *HR*, hazard ratio; *95%CI*, 95% confidence interval.

The association between METS-VF and AF recurrence was further examined using RCS analysis. As shown in **Figure 3**, a linear and positive dose–response relationship was observed between METS-VF and the risk of AF recurrence. The overall association was statistically significant (*p* = 0.02); however, no evidence of non-linearity was detected (*p* = 0.62). This suggests that higher METS-VF scores are linearly associated with an increased risk of AF recurrence following catheter ablation.

## Discussion

To our knowledge, this is the first study that evaluated the association between METS-VF and AF recurrence following catheter ablation. It was found that METS-VF is a strong independent predictor of AF recurrence, with a clear dose-dependent increase in recurrence risk observed across the METS-VF quartiles. This association remained significant after adjusting for conventional clinical and metabolic confounders. Although obesity is a well-established risk factor for AF (12), previous studies have reported the so-called obesity paradox, where individuals with obesity (as defined by their BMI) sometimes exhibit better prognoses compared with their lean counterparts (13). This paradox may have stemmed from the limitations of BMI, which does not distinguish between fat distribution types. Unlike general obesity measured using BMI, visceral adiposity (VAT-driven obesity) is ectopically deposited around critical organs such as the heart, liver, and skeletal muscle (14, 15), contributing to metabolic dysregulation, insulin resistance, and systemic inflammation—all of which are pathophysiological pathways that promote AF substrate formation. Direct VAT quantification through CT or MRI is often impractical in clinical practice due to cost, radiation exposure, and operator dependency (6, 7). Therefore, surrogate indices such as LAP, CMI, and METS-VF have been proposed for the estimation of VAT in a noninvasive and accessible manner (16). Of these, METS-VF offers a distinct advantage as it integrates anthropometric measurements (BMI and waist circumference), lipid profiles, and fasting glucose, capturing the composite metabolic burden more comprehensively than LAP or CMI alone. METS-VF, as a new surrogate used to estimate VAT, is useful for the evaluation of cardiometabolic health and has shown better performance in estimating VAT compared with other surrogates (17, 18). Kapoor et al. validated METS-VF as a reliable, easily available, and inexpensive surrogate for the measurement of VAT; thus, METS-VF exhibited correlations with different diseases such as metabolic disease, cardiovascular disease, and chronic kidney disease (19–22).

In this study, METS-VF demonstrated superior short-term predictive performance for AF recurrence compared with LAP, CMI, and METS-IR, as evidenced by the higher AUC values in the time-dependent ROC analysis and the statistically significant differences confirmed by DeLong’s test. This finding highlights the clinical utility of METS-VF in early post-ablation risk stratification. Furthermore, the RCS analysis revealed a linear dose–response relationship between METS-VF and the risk of AF recurrence, without evidence of a nonlinear threshold effect. This suggests that even moderate increases in METS-VF may incrementally elevate the risk of recurrence. Such a continuous risk gradient supports the use of METS-VF not only as a categorical stratification tool but also as a quantitative biomarker for individualized recurrence risk prediction.

Mechanistically, the components of METS-VF—specifically the markers of insulin resistance, adiposity, and dyslipidemia—may collectively contribute to atrial structural and electrical remodeling, increased atrial fibrosis, and enhanced arrhythmogenic substrate formation. Insulin resistance, a key metabolic abnormality reflected by an elevated METS-VF, has been linked to impaired atrial energy metabolism, increased oxidative stress, and enhanced fibrotic signaling through TGF-β1 pathways (23). These changes can lead to mitochondrial dysfunction and abnormal calcium handling in atrial myocytes, ultimately shortening the action potential duration and facilitating arrhythmogenesis (24). Visceral adiposity, particularly epicardial adipose tissue (EAT), has been shown to exert paracrine effects on the adjacent atrial myocardium, releasing pro-inflammatory cytokines (e.g., TNF-α), adipokines (e.g., leptin and resistin), and fibrotic mediators that promote atrial fibrosis, conduction slowing, and electrical dispersion (25). Recent imaging studies have demonstrated that EAT infiltration is associated with increased low-voltage areas and high recurrence after catheter ablation (26, 27). Recent studies have also suggested that adipose tissue-derived extracellular vesicles (EVs) can carry microRNAs and pro-fibrotic proteins that directly modulate atrial gene expression, indicating a potential epigenetic link between metabolic status and AF substrate progression (28). These interlinked metabolic pathways provide a plausible biological explanation for the observed association. Given its ease of calculation, cost-effectiveness, and predictive capability, METS-VF may serve as a practical tool for clinicians to identify patients at higher risk of AF recurrence after catheter ablation, facilitating personalized follow-up strategies and potential early interventions.

### Study limitation

This study has several limitations. Firstly, this is a retrospective study based on patients who were referred for AF ablation in a single center, which needs validation in other populations. Secondly, the mechanisms between VAT and AF recurrence were not fully understood, and more basic research is needed to investigate these.

## Conclusion

In this study, METS-VF, a reliable surrogate reflecting VAT, was positively associated with increased AF recurrence after ablation. METS-VF could be used to screen those individuals with an increased risk of AF recurrence in clinical practice.

## Data Availability

The raw data supporting the conclusions of this article will be made available by the authors, without undue reservation.
